# PACSIN1 regulates the dynamics of AMPA receptor trafficking

**DOI:** 10.1038/srep31070

**Published:** 2016-08-04

**Authors:** Jocelyn Widagdo, Huaqiang Fang, Se Eun Jang, Victor Anggono

**Affiliations:** 1 Clem Jones Centre for Ageing Dementia Research, Queensland Brain Institute, The University of Queensland, Brisbane, QLD 4072, Australia; 2 Solomon H. Snyder Department of Neuroscience, Johns Hopkins University School of Medicine, Baltimore, MD 21205, USA

## Abstract

Dynamic trafficking of AMPA receptors (AMPARs) into and out of synapses plays an important role in synaptic plasticity. We previously reported that the protein kinase C and casein kinase II substrate in neurons (PACSIN) forms a complex with AMPARs through its interaction with the protein interacting with C-kinase 1 (PICK1) to regulate NMDA receptor (NMDAR)-induced AMPAR endocytosis and cerebellar long-term depression. However, the molecular mechanism by which PACSIN regulates the dynamics of AMPAR trafficking remains unclear. Using a pH-sensitive green fluorescent protein, pHluorin, tagged to the extracellular domain of the GluA2 subunit of AMPARs, we demonstrate dual roles for PACSIN1 in controlling the internalization and recycling of GluA2 after NMDAR activation. Structure and function analysis reveals a requirement for the PACSIN1 F-BAR and SH3 domains in controlling these NMDAR-dependent processes. Interestingly, the variable region, which binds to PICK1, is not essential for NMDAR-dependent GluA2 internalization and is required only for the correct recycling of AMPARs. These results indicate that PACSIN is a versatile membrane deformation protein that links the endocytic and recycling machineries essential for dynamic AMPAR trafficking in neurons.

AMPA receptors (AMPARs) are the main ionotropic glutamate receptors that mediate fast excitatory neurotransmission in the mammalian central nervous system. They are highly mobile and traffic into and out of synapses to regulate synaptic plasticity, a cellular correlate of learning and memory^1^. AMPARs traffic rapidly between intracellular compartments and the plasma membrane via receptor endocytosis, endosomal trafficking, recycling and exocytosis, all of which are differentially regulated in multiple forms of synaptic plasticity^2^. In general, a net increase in AMPAR forward trafficking toward the plasma membrane and synapses results in long-term potentiation. In contrast, removal of AMPARs from the cell surface leads to a decrease in synaptic strength and long-term depression (LTD).

AMPAR trafficking is tightly regulated by a number of intracellular AMPAR interacting proteins, including the BAR (Bin/amphiphysin/Rvs) and PDZ (PSD-95/Dlg/ZO1) domain-containing protein PICK1 (protein interacting with C-kinase 1)^3^,^4^. PICK1, through its PDZ domain, directly binds to the carboxyl tails of GluA2 and GluA3 subunits of AMPARs and plays crucial roles in regulating the surface expression, trafficking and synaptic clustering of AMPARs^4^,^5^,^6^,^7^. Although the involvement of PICK1 in synaptic plasticity, particularly hippocampal and cerebellar LTD, is well established^8^,^9^,^10^,^11^, its underlying mechanism of action remains controversial. Earlier studies proposed a role for PICK1 in the facilitation of AMPAR endocytosis during LTD^11^,^12^,^13^,^14^,^15^. However, more recent evidence suggests that PICK1 is involved in the intracellular retention and recycling of AMPARs^16^,^17^,^18^,^19^,^20^.

The protein kinase C and casein kinase II substrate in neurons (PACSIN), also known as syndapin, is an F-BAR (elongated BAR) and SH3 (src homology-3) domain-containing protein that is capable of remodeling the plasma membrane and mediating protein-protein interactions^21^. It is well established that PACSIN plays important roles in regulating activity-dependent endocytosis and the recycling of presynaptic vesicles^22^,^23^,^24^,^25^,^26^, as well as postsynaptic AMPA, NMDA and glycine receptors^27^,^28^,^29^. Previously, we found that PACSIN interacts with PICK1 to regulate the activity-dependent removal of AMPARs from the plasma membrane^28^. More importantly, loss of PACSIN function or inhibition of the PACSIN−PICK1 interaction led to an impairment in cerebellar LTD^28^. However, it remains unclear how PACSIN regulates the dynamics of AMPAR internalization and recycling following NMDA receptor (NMDAR) activation. Here, we performed molecular replacement analysis and determined the structure and function of various PACSIN mutants in relation to the trafficking of AMPARs in living hippocampal neurons that express the pH-sensitive green fluorescent protein (pHluorin)-tagged GluA2 (pH-GluA2) optical reporter.

## Results and Discussion

### PACSIN is required for AMPAR endocytosis and recycling after NMDAR activation

**Figure 1:**
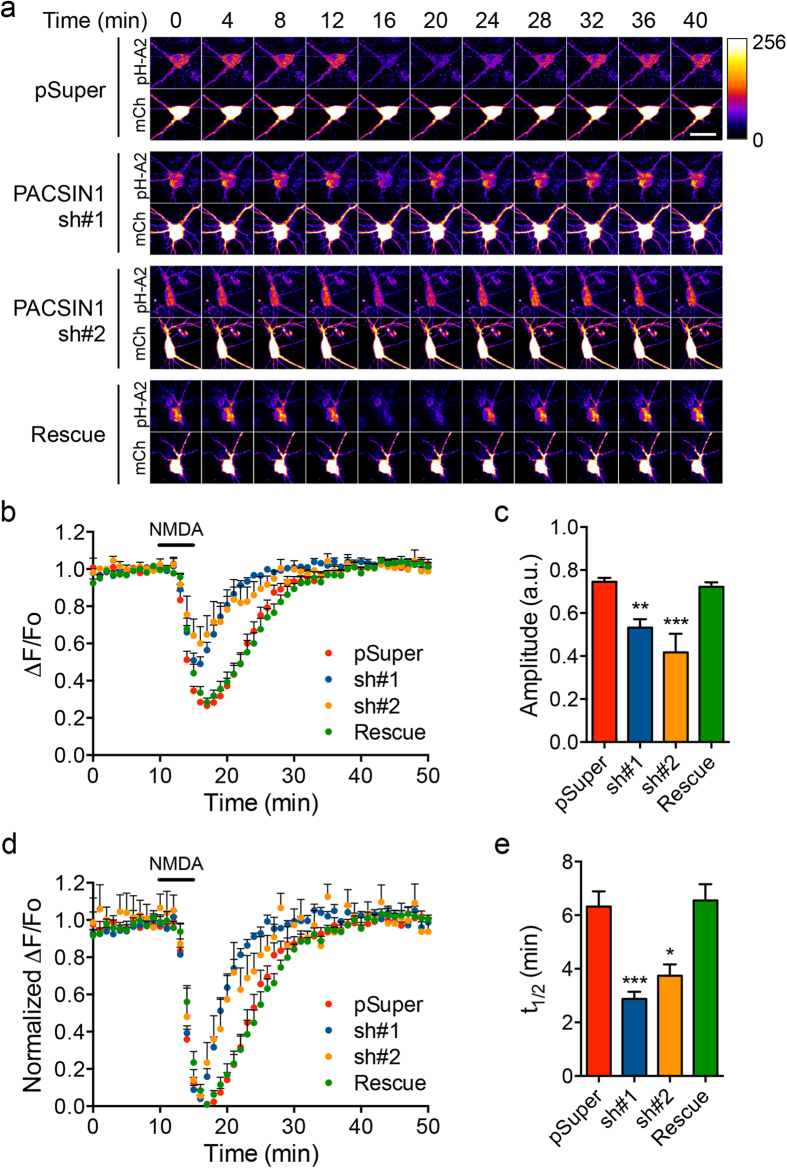
PACSIN1 knockdown inhibits the NMDA-induced internalization of GluA2 and accelerates its rate of recycling to the plasma membrane. Cultured hippocampal neurons were transfected with empty pSuper vector (control), pSuper-PACSIN1-shRNA#1, pSuper-PACSIN1-shRNA#2, or pRK5-H1-shRNA#1-CMV-HA-PACSIN1 rescue constructs together with the pH-GluA2 reporter and mCherry plasmids at DIV15. At DIV17, neurons were stimulated with 20 μM NMDA for 3 min and changes in pH-GluA2 fluorescence intensity were monitored by live-cell confocal microscopy. (**a**) Representative time-series images from control, PACSIN1 knockdown and PACSIN1 rescued neurons (scale bar, 20 μm). Average time course of pH-GluA2 fluorescence changes (ΔF/F_o_) (**b**) and their normalized responses (**d**) in the somatodendritic area. Quantification of the amplitude of pH-GluA2 fluorescence change in response to NMDA stimulation (**c**) and its recycling rate (t_1/2_) after NMDA washout (**e**). Data represent mean ± s.e.m. (One-way ANOVA, *P < 0.05, **P < 0.01, ***P < 0.001, *n* = 10 [pSuper], 12 [sh#1], 7 [sh#2], 13 [rescue] neurons from 4 independent cultures).

We have previously used the conventional antibody-feeding technique to demonstrate that shRNA-mediated knockdown of the neuron-specific PACSIN1 reduces the number of internalized GluA2-containing AMPARs 15 min post-NMDA stimulation^28^. However, we could not rule out a role of PACSIN1 in AMPAR recycling because the accumulation of intracellular receptors measured using this assay reflects the balance between the amount of receptor internalization from the plasma membrane and the recycling of these receptors back to the cell surface. In order to study PACSIN1 function in controlling the dynamics of AMPAR internalization and recycling, we took advantage of pH-GluA2 to visualize the trafficking of AMPARs with high temporal resolution in living hippocampal neurons^19^,^30^,^31^. This reporter was constructed by fusing pHluorin to the extracellular domain of the GluA2 subunit. When endocytosis occurs, the fluorescence of pH-GluA2 initially decreases due to the quenching of pHluorin by the acidic environment inside the lumen of endosomes, but subsequently recovers as pH-GluA2 recycles back to the plasma membrane. In control neurons transfected with pSuper empty vector, perfusion of 20 μM NMDA (in the presence of 1 μM tetrodotoxin) for 3 min resulted in a significant quenching of pH-GluA2 fluorescence in the soma and dendrites due to receptor internalization, which fully recovered within 30 min upon NMDA washout (Fig. 1a,b). The decrease of pH-GluA2 fluorescence intensity was not due to the leakage of protein due to neuronal damage as we never observed any change in cytosolic mCherry fluorescence in the recorded neurons (Fig. 1a). However, the amount of internalized pH-GluA2 was significantly reduced in neurons that overexpressed two independent PACSIN1 shRNA sequences compared with control neurons (Fig. 1a–c). In addition to the endocytosis defect, the rate of pH-GluA2 recycling following NMDA washout was significantly accelerated in PACSIN1 knockdown neurons (Fig. 1d,e). The impairment in NMDA-induced pH-GluA2 internalization and recycling caused by PACSIN1 shRNA#1 could be fully rescued by the overexpression of shRNA#1-resistant PACSIN1 in hippocampal neurons (Fig. 1b–e). Overexpression of PACSIN1 alone did not affect the dynamics of AMPAR trafficking after NMDAR activation (Fig. S1a–d). Furthermore, to ascertain that the effect of PACSIN1 knockdown was specific for pH-GluA2, we examined the response of cytoplasmic pHluorin following NMDA treatment, which is also known to induce acidification of the intracellular compartment^32^. Unlike pH-GluA2, the kinetics of cytosolic pHluorin were indistinguishable between neurons transfected with vector only, PACSIN1 shRNA#1 or a PACSIN1 overexpression construct (Fig. S2a–c). Moreover, the internalization of pH-GluA2 is effectively blocked by two independent endocytosis inhibitors, dynasore and dynole (Fig. S3). This result suggests that the altered kinetics of pH-GluA2 in PACSIN1 knockdown neurons are likely to be due to the trafficking of AMPARs rather than NMDA-induced intracellular acidification. PACSIN1 therefore plays dual roles in controlling the endocytosis and recycling of AMPARs following NMDA stimulation.

### Structure and function analysis of PACSIN in regulating AMPAR dynamics

**Figure 2:**
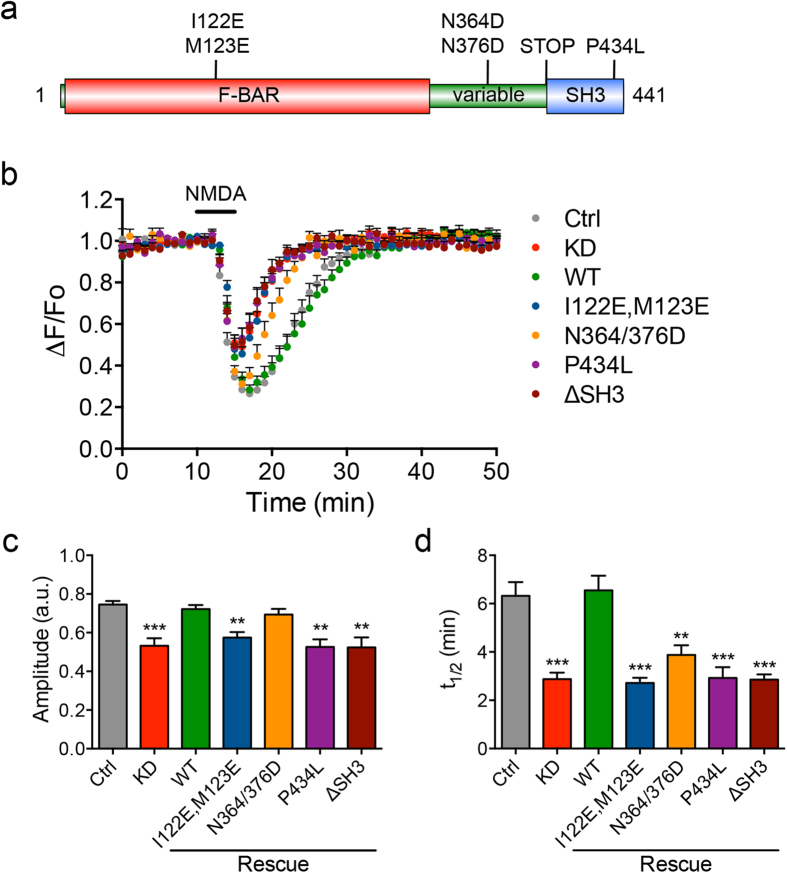
Structure and function analysis of PACSIN1 reveals distinct roles of its functional domains in regulating NMDAR-dependent AMPAR trafficking. (**a**) A schematic diagram of the domain structure of PACSIN1 depicting the location of various mutations used in the molecular replacement study. (**b**) Average time course of pH-GluA2 fluorescence changes (ΔF/F_o_) and their normalized responses in PACSIN1 knockdown and rescued neurons expressing various mutations. Quantification of the amplitude of pH-GluA2 fluorescence change in response to NMDA stimulation (**c**) and its recycling rate (t_1/2_) after NMDA washout (**d**). Data represent mean ± s.e.m. (One-way ANOVA, **P < 0.01, ***P < 0.001, *n* = 10 [control], 12 [knockdown], 13 [WT], 15 [I122E,M123E], 8 [N346/376D], 9 [P434L], 8 [ΔSH3] neurons from 6 independent cultures).

Next, we sought to understand the underlying mechanism of action by performing structure and function analysis of PACSIN1. Using a molecular replacement strategy, we determined the molecular requirement for each functional domain of PACSIN1 in regulating NMDAR-dependent AMPAR endocytosis and recycling (Fig. 2a). Recent structural studies have identified a unique loop of hydrophobic residues (Ile-122 and Met-122) that protrude from the membrane interaction surface of the PACSIN1 F-BAR domain^33^. This so-called “wedge-loop” is believed to be inserted into a membrane leaflet to promote membrane bending and is thought to contribute to the formation of budding vesicles from the plasma membrane during endocytosis. Replacing these two hydrophobic residues with negatively charged amino acids completely abolishes the ability of the PACSIN1 F-BAR domain to bind lipids and tubulate liposomes *in vitro*^33^,^34^,^35^. In contrast to PACSIN1 wild-type (WT) expression, replacing endogenous PACSIN1 with the I122E,M123E wedge-loop mutant failed to rescue the pH-GluA2 endocytosis and recycling deficit in PACSIN1 knockdown neurons (Fig. 2b–d). These data demonstrate that the membrane binding and deformation activities of the PACSIN1 F-BAR domain are critical for the activity-dependent trafficking of AMPARs following NMDAR activation in neurons.

The SH3 domain of PACSIN1 is a well-known protein-protein interaction module that binds to the large GTPase dynamin and the actin cytoskeleton regulator, N-WASP (neural Wiskott-Aldrich syndrome protein), both of which play crucial roles in endocytosis^36^. To determine the importance of the PACSIN1 SH3 domain, we examined the effect of the P434L mutant, which disrupts all PACSIN1 SH3-dependent interaction with its proline-rich domain-containing binding partners, including dynamin and N-WASP. We found that substituting endogenous PACSIN1 with the P434L mutant also caused impairment in AMPAR endocytosis and recycling (Fig. 2b–d). Similarly, deletion of the entire SH3 domain (PACSIN1 ΔSH3) phenocopied the effect of the PACSIN1 P434L mutant (Fig. 2b–d). Given the established involvement of dynamin and the actin cytoskeleton in AMPAR trafficking^37^,^38^,^39^, these results indicate a potential role of PACSIN1 as a scaffold protein linking dynamin and actin polymerization to promote vesicle fission and provide mechanical forces to propel these vesicles away from the plasma membrane.

Next, we investigated the effect of the PACSIN1 N364/376D mutation in the NPF motifs within the variable region, which is known to disrupt interaction with Eps15 homology domain (EHD) protein 1, also known as Rme1^40^, a crucial regulator of AMPAR recycling in neurons^41^. Interestingly, the PACSIN1 N364/376D mutant displayed comparable endocytosis to pH-GluA2 but an accelerated rate of reinsertion after NMDA washout (Fig. 2b–d). This result demonstrates that the PACSIN1−EHD1 interaction is not essential for NMDA-induced GluA2 endocytosis, but plays a pivotal role in GluA2 recycling.

### The PICK1**−**PACSIN interaction is specifically required for the NMDAR-dependent recycling of AMPARs

**Figure 3:**
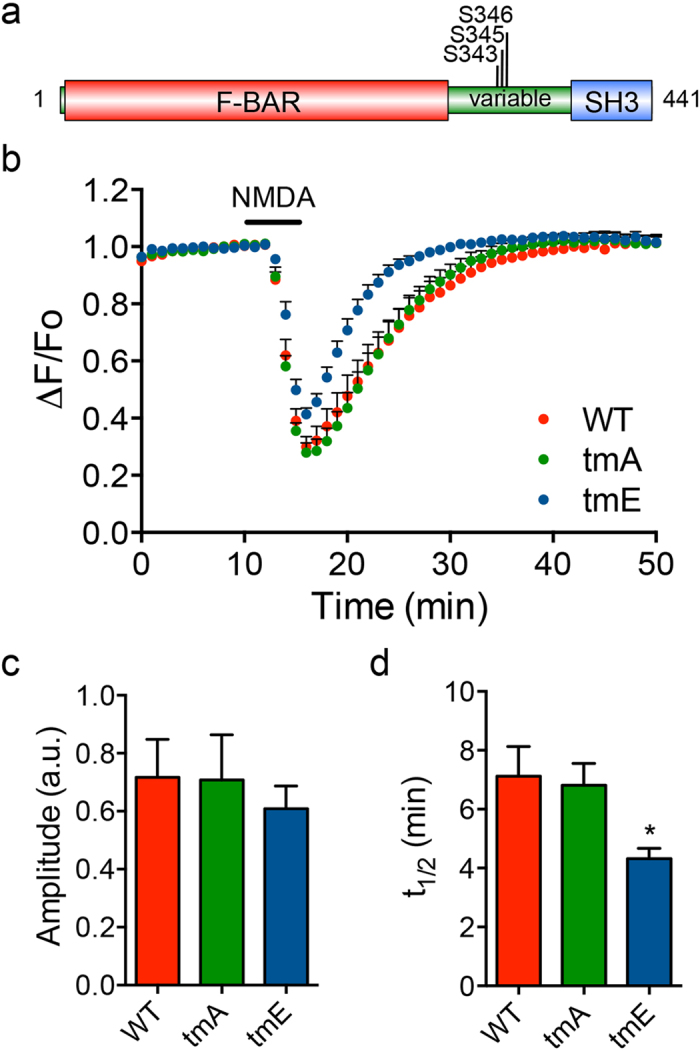
PACSIN1 phosphomimetic mutation in the variable region alters the rate of AMPAR recycling. (**a**) A schematic diagram of PACSIN1 showing the phosphorylation sites in the variable region. (**b**) Average time course of NMDA-induced pH-GluA2 fluorescence changes (ΔF/F_o_) in neurons expressing either PACSIN1 WT, a tmA (S343A, S345A and S346A) or tmE (S343E, S345E and S346E) phosphomutant. Quantification of the amplitude of pH-GluA2 fluorescence change in response to NMDA stimulation (**c**) and its recycling rate (t_1/2_) after NMDA washout (**d**). Data represent mean ± s.e.m. (One-way ANOVA, *P < 0.05, *n* = 13 (WT), 15 (tmA), 16 (tmE) neurons from 3 independent cultures).

The variable region of PACSIN1 is also known to interact with PICK1, although the binding is independent of NPF motifs^28^. To assess the role of the PICK1−PACSIN1 interaction in the dynamic trafficking of AMPARs, we replaced endogenous PACSIN1 with the PACSIN1 tmE mutant, in which Ser-343, Ser-345 and Ser-346 are mutated to glutamates, and which displays reduced binding to PICK1^28^ (Fig. 3a). The expression of the PACSIN1 tmE mutant did not affect NMDA-induced pH-GluA2 internalization, but produced an increased rate of recycling (Fig. 3b–d). In contrast, the effect of the PACSIN1 tmA (alanine) mutant was indistinguishable from that of PACSIN1 WT (Fig. 3b–d). These data suggest that the PACSIN1−PICK1 interaction is specifically required to regulate the rate of GluA2 recycling.

**Figure 4:**
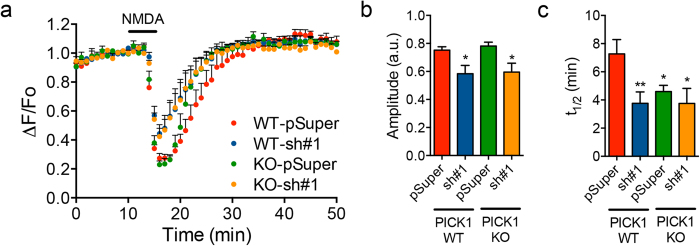
PICK1–PACSIN1 interaction regulates AMPAR recycling following NMDA stimulation. Cultured cortical neurons derived from PICK1 WT and knockout (KO) mice and transfected with empty pSuper vector (control) or pSuper-PACSIN1-shRNA#1 were subjected to the pH-GluA2 recycling assay. (**a**) Average time course of pH-GluA2 fluorescence changes (ΔF/F_o_) in neurons. Quantification of the amplitude of the pH-GluA2 fluorescence change in response to NMDA stimulation (**b**) and its recycling rate (t_1/2_) after NMDA washout (**d**). Data represent mean ± s.e.m. (One-way ANOVA, *P < 0.05, **P < 0.01, *n* = 7 (PICK1-WT/pSuper), 7 (PICK1-WT/sh#1), 6 (PICK1-KO/pSuper), 6 (PICK1-KO/sh#1) neurons from 3 independent cultures).

To further confirm these results, we assessed the effect of PACSIN1 knockdown in primary neurons derived from either PICK1 WT or knockout mice. PICK1 knockout neurons displayed normal endocytosis of pH-GluA2, but an accelerated rate of recycling upon NMDA washout (Fig. 4a–d), consistent with a role of PICK1 in regulating AMPAR recycling by retaining internalized GluA2 in endosomes^16^,^17^,^18^,^19^,^20^. We found that PACSIN1 knockdown was able to reduce the amplitude of pH-GluA2 endocytosis in both PICK1 WT and knockout neurons (Fig. 4a,b), suggesting that the role of PACSIN1 in regulating AMPAR endocytosis is independent of PICK1 function. However, loss of PACSIN1 function in PICK1 knockout neurons did not further enhance the rate of pH-GluA2 recycling (Fig. 4a,b), indicating that both PICK1 and PACSIN1 regulate pH-GluA2 recycling through a common pathway.

**Figure 5:**
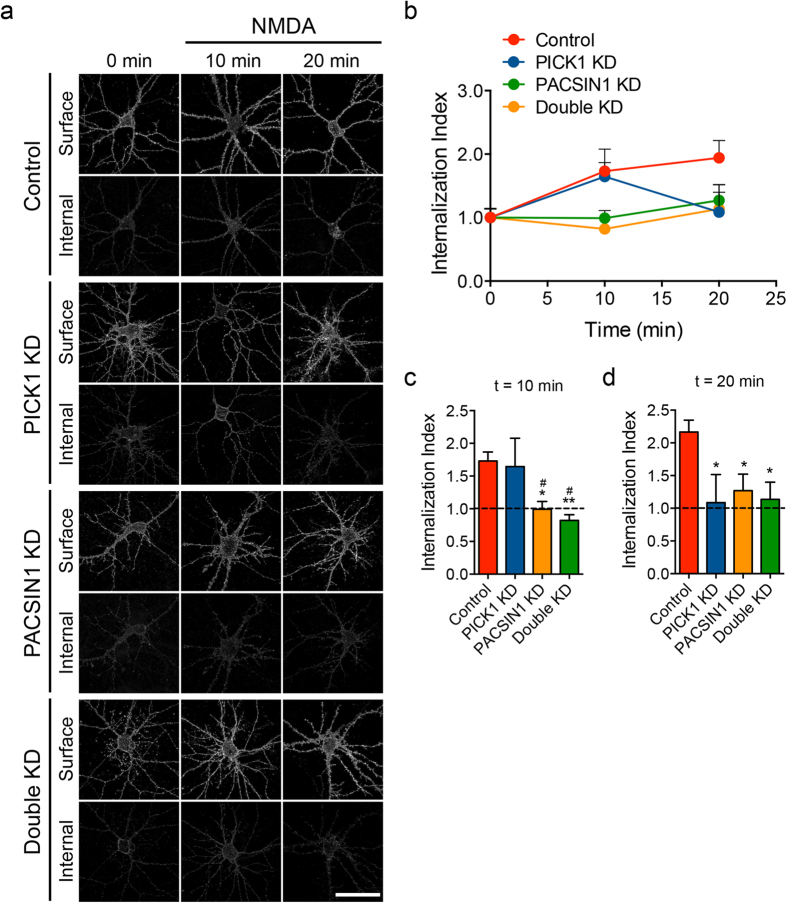
The role of PACSIN1 in regulating NMDA-induced AMPAR endocytosis is independent of PICK1. (**a**) Cultured hippocampal neurons were transfected with empty pSuper vector (control), pSuper-PICK1-shRNA (PICK1 KD), pSuper-PACSIN1-shRNA#1 (PACSIN1 KD) or a combination of PICK1 and PACSIN1 shRNA constructs (double KD) together with the myc-GluA2 reporter at DIV15. At DIV17, neurons were stimulated with 50 μM NMDA for 5 min and the extent of myc-GluA2 internalization was monitored 5 min (t = 10 min) and 15 min (t = 20 min) after stimulation. Changes in pH-GluA2 fluorescence intensity were monitored by live-cell confocal microscopy (scale bar, 50 μm). Quantification of myc-GluA2 internalization at different time points (**b**), at t = 10 min (**c**) and at t = 20 min (**d**). Data represent mean ± s.e.m. (One-way ANOVA, *P < 0.05, **P < 0.01 against control cells, ^#^*P* < 0.05 against PICK1 KD cells, *n* = 8 (Control), 6 (PICK1 KD), 6 (PACSIN1 KD), 6 (double KD) neurons for each time point from 2 independent cultures).

To confirm that PACSIN1 regulates the NMDA-induced internalization of AMPARs independent of PICK1, we performed an antibody-feeding assay with myc antibodies to measure the degree of intracellular accumulation of endocytosed myc-GluA2 subunits in live transfected hippocampal neurons over time. In control neurons, myc-GluA2 internalization could be observed at 10 min time point after a 5 min treatment with NMDA (50 μM NMDA + 1 μM tetrodotoxin), with the remaining myc-GluA2 in the intracellular compartment over the next 10 min (Fig. 5a–d). In PICK1 knockdown neurons, the same proportion of surface myc-GluA2 was internalized 10 min after NMDA treatment (Fig. 5a–d). Consistent with the role of PICK1 in prolonging intracellular retention of AMPARs^16^,^17^,^18^,^19^,^20^, these internalized receptors fully recycled back to the plasma membrane within the next 10 min (Fig. 5a–d). Importantly, loss of PACSIN1 function caused a significant reduction in myc-GluA2 internalization in PICK1 wild-type and knockdown neurons (Fig. 5a–d). These results provide an independent confirmation to support our previous findings.

**Figure 6:**
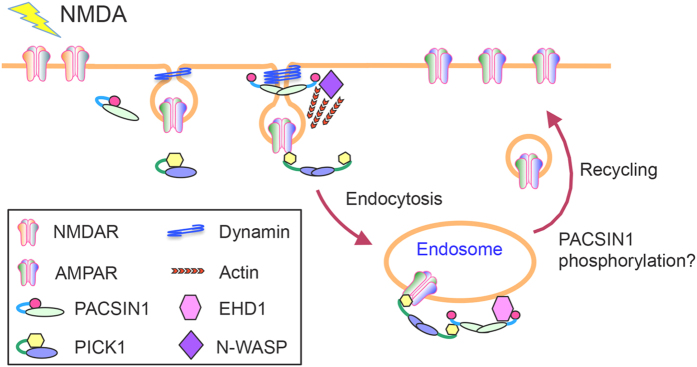
Proposed model for the role of PACSIN1 in controlling AMPAR endocytosis and recycling. In this model, activation of NMDARs triggers the recruitment of PACSIN1 to the clathrin-coated pit at the plasma membrane through an SH3-dependent interaction with a component of the endocytic machinery, such as dynamin or N-WASP. This endocytic complex facilitates membrane deformation, invagination and fission to generate vesicles containing AMPARs, as well as actin nucleation to provide mechanical forces to propel vesicles away from the plasma membrane. Subsequent interaction of PACSIN1 with EHD1 and/or PICK1 controls the intracellular retention and the correct recycling of AMPARs to the plasma membrane. We propose that the activity-dependent phosphorylation of PACSIN1 in the variable region serves as a molecular switch that regulates the efficient recycling of AMPARs to support synaptic transmission and plasticity.

In conclusion, we have demonstrated dual roles of PACSIN1 in controlling the NMDAR-dependent dynamics of AMPAR endocytosis and recycling that involves multiple regulatory elements through its distinct functional domains. We propose that, during AMPAR endocytosis, PACSIN1 is recruited to the clathrin-coated pit at the plasma membrane through a SH3-dependent interaction with a component of the endocytic machinery, such as dynamin (Fig. 6). The binding of dynamin to the SH3 domain forces PACSIN1 into an open conformation and releases the intramolecular inhibition of the PACSIN1 F-BAR domain, which is essential for its membrane deformation activity and facilitates vesicle endocytosis and fission. Given that PACSIN1 exists in dimers and oligomers, the other PACSIN1 SH3 domain can interact with the actin nucleator, N-WASP, to provide mechanical forces to propel vesicles away from the plasma membrane through active actin remodeling. Subsequently, PACSIN1 could bind to EHD1 and/or PICK1 to control the intracellular retention and correct recycling of AMPARs to the plasma membrane. The fact that the PACSIN1-PICK1 interaction can be tuned, potentially by phosphorylation of multiple serine residues in the variable region, adds another layer of regulation to ensure that AMPARs are recycled efficiently and in a timely manner to support synaptic transmission and plasticity.

## Methods

### DNA constructs

The PACSIN1 shRNA targeting sequences and the bicistronic pRK5-H1-shRNA#1-CMV-HA-PACSIN1 rescue construct were generated and characterized previously^24^,^28^. PACSIN1 point mutants were generated using the overlapping PCR protocol. Plasmids encoding PICK1 shRNA, myc-GluA2, pH-GluA2 and cytosolic pHluorin have been reported previously^18^,^28^,^32^,^42^.

### Neuronal cultures and transfection

All animal handling procedures were approved by the Animal Care and Use Committee of the Johns Hopkins University School of Medicine and the University of Queensland Animal Ethics Committee, and were conducted in accordance with relevant guidelines and regulations set by the National Institutes of Health in the USA and the Australian National Health and Medical Research Council. Hippocampal neurons from embryonic day 18 rat pups were plated onto poly-L-lysine-coated coverslips in Neurobasal growth medium supplemented with 2% B27, 2 mM Glutamax, 50 U/mL penicillin, 50 μg/mL streptomycin and 5% fetal bovine serum. Neurons were switched to serum-free Neurobasal medium 24 h after seeding and fed twice a week. Homozygote PICK1 knockout mice^43^ were obtained by mating heterozygote parents. High-density cortical neurons were prepared from PICK1 knockout pups and WT littermates at postnatal day 0 as previously described^18^. Neurons were maintained in glial-conditioned Neurobasal medium supplemented with 1% horse serum, 2% B-27 and 2 mM Glutamax, and fed twice a week. Neurons were transfected at days *in vitro* (DIV) 12–14 using lipofectamine 2000 (Invitrogen).

### pH-GluA2 recycling assay

pH-GluA2 recycling live-imaging assays were performed 48 h post-transfection as described previously^42^. Briefly, coverslips containing neurons were assembled in a closed perfusion chamber and continuously perfused with artificial cerebrospinal fluid (ACSF; 25 mM HEPES, 120 mM NaCl, 5 mM KCl, 2 mM CaCl_2_, 2 mM MgCl_2_, 30 mM D-glucose, 1 μM tetrodotoxin, pH 7.4). After 10 min of baseline recording (F_0_), neurons were perfused with NMDA solution (25 mM HEPES, 120 mM NaCl, 5 mM KCl, 2 mM CaCl_2_, 0.3 mM MgCl_2_, 30 mM D-glucose, 1 μM tetrodotoxin, 20 μM NMDA, 10 μM glycine, pH 7.4) for 3 min before the perfusion medium was switched back to recording buffer for the remainder of the session. All imaging experiments were performed at room temperature using a Zeiss LSM 510 Meta/NLO system. The pHluorin fluorescence was imaged at 488 nm excitation and collected through a 505–550 nm filter, while the mCherry signal was imaged at 561 nm excitation and 575–615 nm emission. Neurons were imaged through a 63X oil objective (N.A. = 1.40) at a 3 μm single optical section close to the plasma membrane and collected at a rate of 1 image per min. Images were analyzed using ImageJ software (NIH) by calculating the normalized change in average pHluorin over mCherry fluorescence intensities from a somatodendritic area defined manually to compensate for x-y movements of the recorded neurons. The fluorescence intensity change is expressed as ΔF/F_o_ and the amplitude of fluorescence change (ΔF_max_/F_o_) represents the extent of GluA2 endocytosis. The rate of GluA2 recycling can be calculated as the time taken from fluorescence minima to 50% of the fluorescence maxima (t_1/2_).

### Antibody-feeding myc-GluA2 internalization assays

Cultured hippocampal neurons were transfected with PICK1 and PACSIN1 knockdown constructs, together with a myc-GluA2 reporter construct. To determine the amount of receptor internalization, surface myc-GluA2 was first labeled with mouse anti-myc antibody in live neurons, followed by 5 min incubation with 50 μM NMDA + 1 μM tetrodotoxin, prepared in normal ACSF containing 2 mM Mg^2+^, to induce receptor internalization. Cells were fixed with 4% paraformaldehyde/4% sucrose in PBS 5 min (t = 10′) or 15 min (t = 20′) after NMDA treatment. The remaining surface myc antibody was stained with a saturating concentration of Alexa-568 anti-mouse secondary antibody under non-permeabilizing conditions (surface), and the internalized myc antibody was labeled with Alexa-488 anti-mouse secondary antibody once neurons were permeabilized (internalized). Images were collected with a 63X oil-immersion objective on a Zeiss LSM510 confocal microscope. Fluorescence intensities were quantified using image J software (NIH). Data were expressed as the internalized/total (surface + internal) GluA2 ratio (internalization index).

### Statistics

All data sets were subjected to one-way ANOVA statistical test followed by the Tukey’s multiple comparison test calculated using Prism5 (GraphPad). Results were presented as mean ± standard error of the means.

## Additional Information

**How to cite this article**: Widagdo, J. *et al*. PACSIN1 regulates the dynamics of AMPA receptor trafficking. *Sci. Rep.* **6**, 31070; doi: 10.1038/srep31070 (2016).

## Supplementary Material

Supplementary Information
