# Supplementary material for: PACSIN1 regulates the dynamics of AMPA receptor trafficking
Source: Sci Rep. 2016 Aug 4;6:31070. doi: 10.1038/srep31070 (PMC4973260; doi:10.1038/srep31070)
Supplement: Supplementary Information [file srep31070-s1.pdf]

SUPPLEMENTAL INFORMATION

PACSIN1 regulates the dynamics of AMPA receptor trafficking

Jocelyn Widagdo[1,*], Huaqiang Fang[2,*], Se Eun Jang[1] and Victor Anggono[1,‡]

[1]Clem Jones Centre for Ageing Dementia Research, Queensland Brain Institute, The University of Queensland, Brisbane, QLD 4072, Australia

[2]Solomon H. Snyder Department of Neuroscience, Johns Hopkins University School of Medicine, Baltimore, MD 21205, USA

[*]These authors contributed equally to this work

[‡]**Corresponding author:**

Dr. Victor Anggono

Clem Jones Centre for Ageing Dementia Research

Queensland Brain Institute

The University of Queensland

Brisbane, QLD 4072, Australia

v.anggono@uq.edu.au

Keywords: AMPA receptors, endocytosis, recycling, PICK1, PACSIN/syndapin.

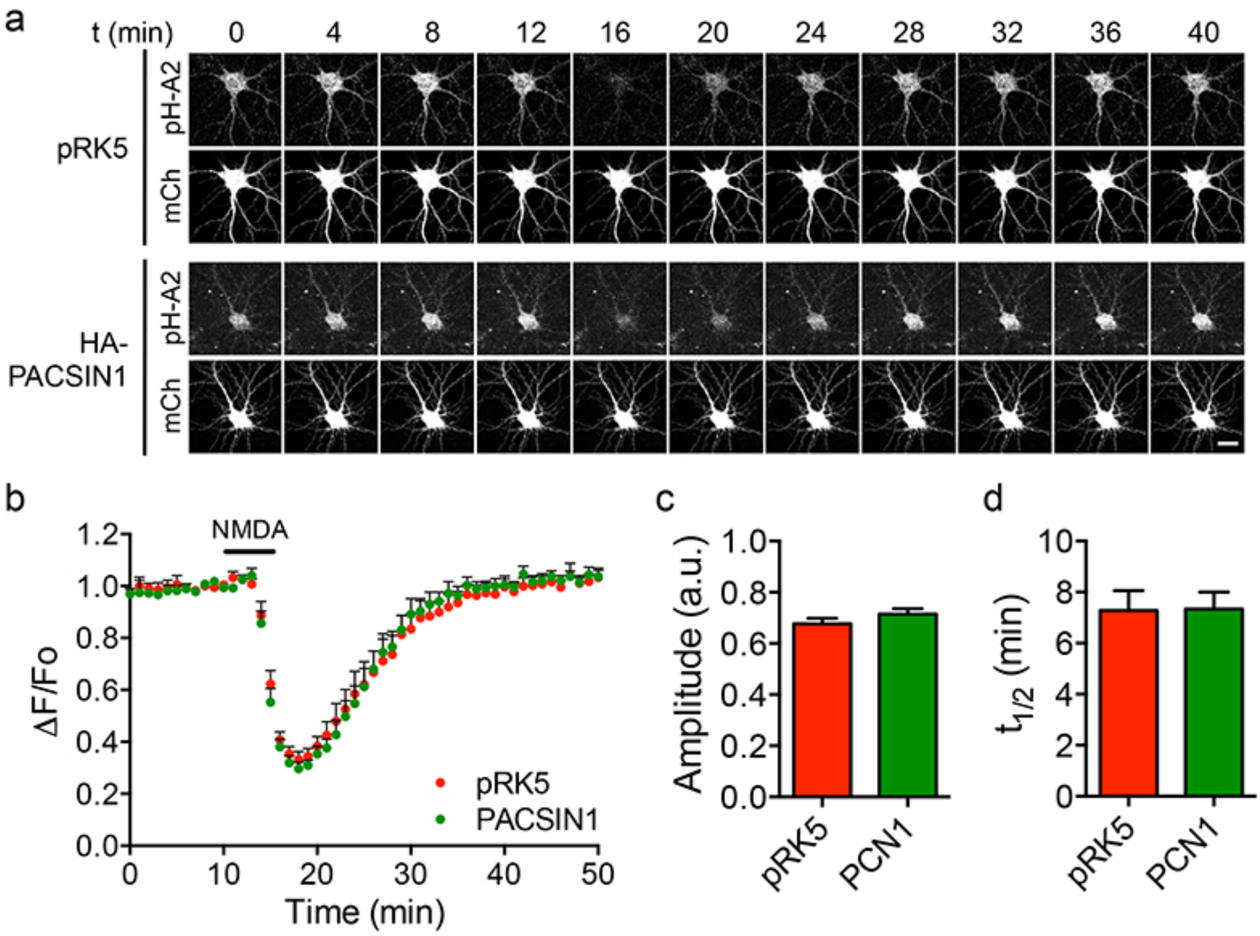

Figure S1. Overexpression of PACSIN1 does not affect NMDA-induced AMPAR endocytosis and recycling in hippocampal neurons. Cultured hippocampal neurons were transfected with empty pRK5 vector (control) or pRK5-HA-PACSIN1 construct together with the pH-GluA2 reporter and mCherry plasmids at DIV15. At DIV17, neurons were stimulated with 20 μM NMDA for 3 min and changes in pH-GluA2 fluorescence intensity were monitored by live-cell confocal microscopy. (**a**) Representative time-series images from control, PACSIN1 knockdown and PACSIN1 rescued neurons (scale bar, 20 μm). (**b**) Average time course of pH-GluA2 fluorescence changes ($\Delta F/F_o$) in the somatodendritic area. Quantification of the amplitude of pH-GluA2 fluorescence change in response to NMDA stimulation (**c**) and its recycling rate ($t_{1/2}$) after NMDA washout (**d**). Data represent mean±s.e.m. *n*=12 (pRK5), 10 (PACSIN1) neurons from 3 independent cultures.

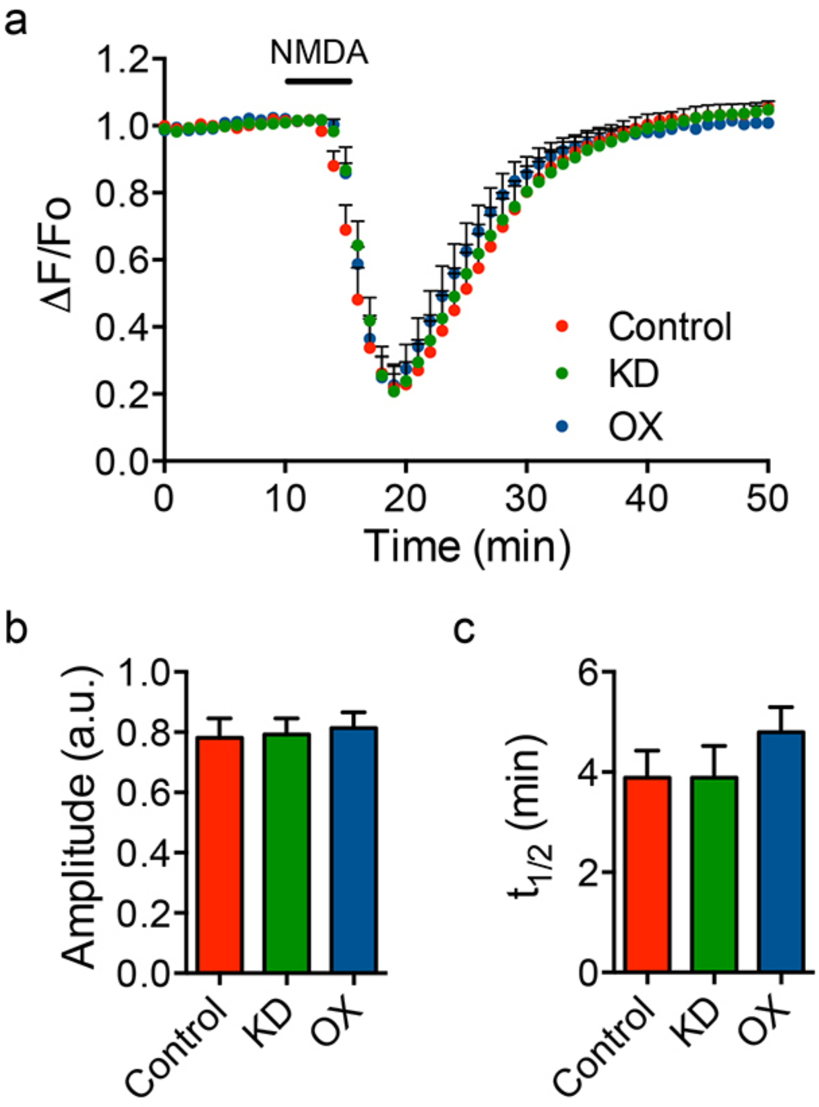

Figure S2. The levels of PACSIN1 expression do not affect the response of cyto-pHluorin to NMDA-induced intracellular acidification. Cultured hippocampal neurons were transfected with empty pSuper vector (control), pRK5-HA-PACSIN1 (OX) or pSuper-PACSIN1-shRNA#1 (KD) constructs together with the cyto-pHluorin reporter and mCherry plasmids at DIV15. At DIV17, neurons were stimulated with 20 μM NMDA for 3 min and changes in cyto-pHluorin fluorescence intensity were monitored by live-cell confocal microscopy. (**a**) Average time course of cyto-pHluorin fluorescence changes ($\Delta F/F_o$) in the somatodendritic area. Quantification of the amplitude of cyto-pHluorin fluorescence quenching (**b**) and its recovery rate ($t_{1/2}$) upon NMDA stimulation (**c**). Data represent mean±s.e.m. *n*=7 (control), 11 (OX) and 12 (KD) neurons from 3 independent cultures.

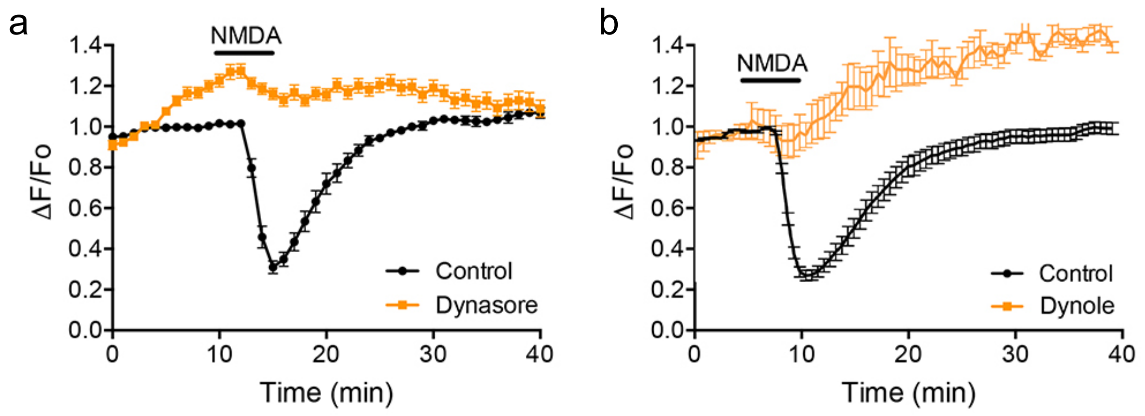

Figure S3. NMDA-induced internalization of pH-GluA2 is blocked by endocytosis inhibitors. Cultured hippocampal neurons were co-transfected with the pH-GluA2 reporter and mCherry plasmids at DIV15. At DIV17, neurons were pre-incubated with 80 μM dynasore (**a**) or 20 μM dynole 34-2 (**b**) for 10 min prior to the recycling assay induced by 20 μM NMDA stimulation for 3 min. Inhibitors were present throughout the imaging session. *n*=13 (control for dynasore), 15 (dynasore), 9 (control for dynole) and 3 (dynole) neurons from 3 independent cultures.
